# Aggregation-induced photocatalytic activity and efficient photocatalytic hydrogen evolution of amphiphilic rhodamines in water†

**DOI:** 10.1039/d0sc04285d

**Published:** 2020-10-07

**Authors:** Hajime Shigemitsu, Youhei Tani, Tomoe Tamemoto, Tadashi Mori, Xinxi Li, Yasuko Osakada, Mamoru Fujitsuka, Toshiyuki Kida

**Affiliations:** Department of Applied Chemistry, Graduate School of Engineering, Osaka University Suita 565-0871 Japan shigemitsu@chem.eng.osaka-u.ac.jp kida@chem.eng.osaka-u.ac.jp; Frontier Research Base for Global Young Researchers, Graduate School of Engineering, Osaka University Suita 565-0871 Japan; Global Center for Medical Engineering and Informatics, Osaka University Suita 565-0871 Japan; The Institute of Scientific and Industrial Research (SANKEN), Osaka University Mihogaoka 8-1 Ibaraki Osaka 567-0047 Japan; Institute for Advanced Co-creation Studies, Osaka University 1-1 Yamadagaoka Suita Osaka 565-0871 Japan

## Abstract

The development of photocatalysts is an essential task for clean energy generation and establishing a sustainable society. This paper describes the aggregation-induced photocatalytic activity (AI-PCA) of amphiphilic rhodamines and photocatalytic functions of the supramolecular assemblies. The supramolecular assemblies consisting of amphiphilic rhodamines with octadecyl alkyl chains exhibited significant photocatalytic activity under visible light irradiation in water, while the corresponding monomeric rhodamines did not exhibit photocatalytic activity. The studies on the photocatalytic mechanism by spectroscopic and microscopic analyses clearly demonstrated the AI-PCA of the rhodamines. Moreover, the supramolecular assemblies of the rhodamines exhibited excellent photocatalytic hydrogen evolution rates (up to 5.9 mmol g^−1^ h^−1^).

## Abstract 2

Simple amphiphilic rhodamines formed supramolecular assemblies and exhibited aggregation-induced photocatalytic activity and hydrogen evolution in water.

## Graphical abstract

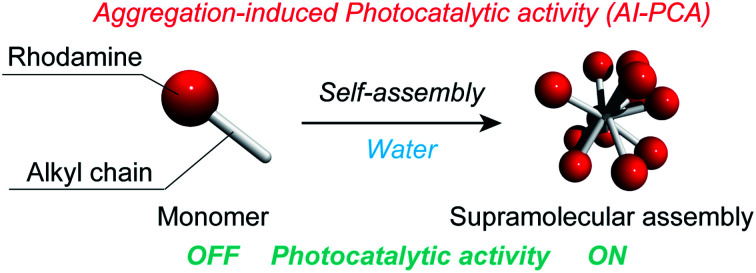

## Introduction

Photocatalysts are promising materials for the conversion of solar energy into storable chemical energy and are expected to contribute significantly to clean and renewable energy generation.^1^ In 1974, Fujishima and Honda reported photocatalytic water-splitting using a titanium dioxide electrode, demonstrating the possibility of artificial photosynthesis.^2^ Since then, a wide range of photocatalysts, based on inorganic,^3^ molecular,^4^ and polymeric^5^ compounds, have been actively developed. Besides their application in artificial photosynthesis, the redox reactivity of photocatalysts has been utilized for environmental remediation,^6^ organic synthesis,^7^ and photodynamic therapy.^8^ The emergence and development of new photocatalysts have contributed to the progress in artificial photosynthesis and generated new opportunities in the related fields.^9^

Based on these backgrounds, we explored a new class of photocatalysts and focused on supramolecular assemblies. The photophysical properties of supramolecular assemblies are different from those of the constituting monomers because of the interaction between the adjacent molecules.^10^ Various characteristic aggregation-induced photophysical phenomena, such as aggregation-caused quenching (ACQ),^11^ aggregation-induced enhanced emission,^12^ light-harvesting,^13^ and nonlinear optical phenomena (*e.g.*, photon upconversion^14^ and singlet fission^15^) have been intensively studied and applied to solar energy collection,^16^ molecular sensing,^17^ and biological applications (*e.g.*, bioimaging,^18^ optogenetics,^19^ and phototherapy^20^). However, aggregation-induced photocatalytic activity (AI-PCA) has never been reported despite the high potential for a novel photocatalytic material. Taking into account previous reports on aggregation-induced triplet excited state generation^21^ and charge carrier migration^22^ in self-assembled nanostructures of organic dyes, we considered that various organic dyes may cause AI-PCA. These phenomena cause elongation of the excited state lifetime^23^ and increasing collision frequency with substrates,^24^ which are important for the progression of photocatalytic reactions. AI-PCA would lead to expansion of the molecular design of photocatalysts that enables adjustment of absorption wavelength and redox potential. In addition, self-assembled supramolecular photocatalysts (SA-SPCs) possessing AI-PCA are expected to produce unprecedented photocatalytic soft-materials^25^ (gel, liquid crystal, membrane *etc.*) taking advantages of the unique properties of supramolecular assemblies^26^ (*e.g.* reversibility and stimuli-responsiveness).

**Fig. 1:**
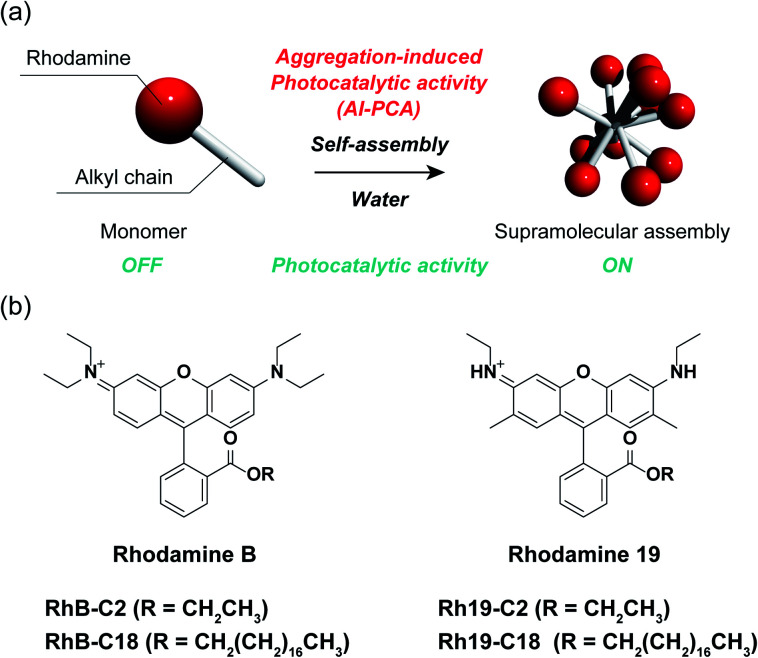
(a) Schematic representation of aggregation-induced photocatalytic activity (AI-PCA). (b) Chemical structures of rhodamine derivatives (left: rhodamine B (**RhB**), and right: rhodamine 19 (**Rh19**)).

Herein, we demonstrate the AI-PCA of amphiphilic rhodamines (Fig. 1a). Rhodamines are very common hydrophilic organic dyes with excellent photophysical properties, such as high light absorption and quantum yield, which can be tuned through chemical modification.^27^ The two SA-SPCs composed of amphiphilic rhodamines (rhodamine B (**RhB**) and rhodamine 19 (**Rh19**) (Fig. 1b)) exhibited photocatalytic activity under visible light irradiation in water, while the monomeric rhodamines did not exhibit photocatalytic activity. In particular, the SA-SPCs exhibited excellent hydrogen evolution rates.

## Results and discussion

### Molecular design of the rhodamine derivatives

Four rhodamine derivatives with short and long alkyl chains were used in this study (Fig. 1b). Two common rhodamines with different absorption bands, **RhB** and **Rh19**, were selected as hydrophilic organic dyes to examine the concept of AI-PCA. The amphiphilic rhodamines with octadecyl alkyl chains (**RhB-C18** and **Rh19-C18**) were expected to form supramolecular assemblies in water through hydrophobic interaction between the alkyl chains. More hydrophilic rhodamine derivatives with an ethyl ester group (**RhB-C2** and **Rh19-C2**) compared to those with octadecyl alkyl chains were prepared as control compounds to evaluate the effect of self-assembly on the photocatalytic activity.

**Fig. 2:**
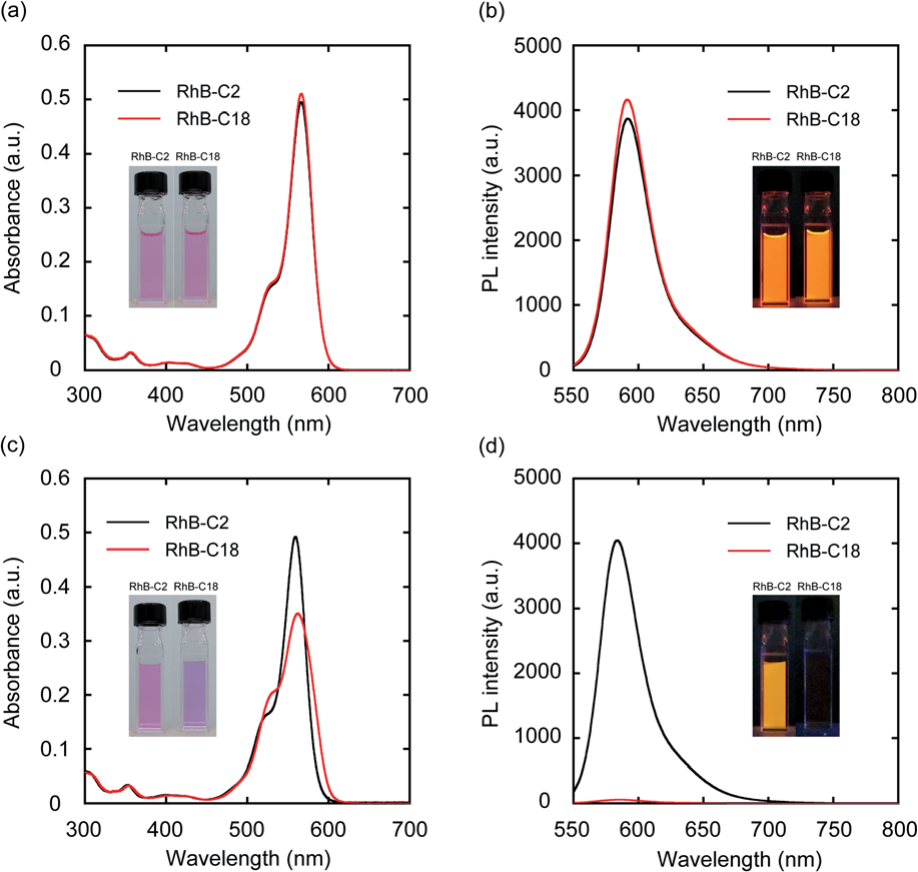
(a, c) UV-vis absorption (UV-vis) and (b, d) photoluminescence (PL) spectra of **RhB-C2** and **RhB-C18** in DMSO (a, b) and water (c, d). Experimental conditions: [**RhB-C2**] = [**RhB-C18**] = 5.0 μM, rt, water, excitation wavelength: 540 nm. Insets: Optical photos of **RhB-C2** and **RhB-C18** in DMSO and water under (a, c) daylight and (b, d) UV light (365 nm).

The photophysical properties of monomeric **RhB-C2** and **RhB-C18** were evaluated from their UV-vis absorption (UV-vis) and photoluminescence (PL) spectra measured in dimethyl sulfoxide (DMSO), which is a good solvent for these compounds (Fig. 2a and b). The absorption spectra of **RhB-C2** and **RhB-C18** corresponded well with each other (Fig. 2a) and both the compounds exhibited absorption maxima at 566 nm. **RhB-C2** and **RhB-C18** also exhibited similar PL spectra with an emission peak at *λ*_em_ = 592 nm (Fig. 2b). These results indicate that the electronic states of **RhB-C2** and **RhB-C18** are quite similar despite the different alkyl chain lengths. We thus conclude that this pair is suitable for evaluating the effect of self-assembly on their photophysical and photocatalytic properties. Further, **Rh19-C2** and **Rh19-C18** also exhibited similar UV-vis and PL spectra (Fig. S1a and b†) with maxima at *λ*_abs_ = 539 nm and *λ*_em_ = 565 nm, respectively, which indicate that the **Rh19-C2/Rh19-C18** pair has similar electronic states regardless of the alkyl chain length.

### Self-assembling properties of the rhodamines in water

To examine the self-assembly properties, the UV-vis and PL spectra of **RhB-C2** and **RhB-C18** were recorded in water (concentration: 5.0 μM) (Fig. 2c and d). The UV-vis spectrum of **RhB-C2** in water is similar to that in DMSO with a slightly red-shifted absorption maximum (*λ*_abs_: 559 nm (DMSO), 562 nm (water)) (Fig. 2c). In contrast, **RhB-C18** exhibited a broad spectrum with split peaks at 530 and 559 nm that can be assigned to the aggregation states of **RhB-C18**,^28^ which suggests the formation of a supramolecular assembly of **RhB-C18** in water. The PL spectra of **RhB-C2** and **RhB-C18** were significantly different (Fig. 2d). **RhB-C2** exhibited an intense emission in water, whereas **RhB-C18** exhibited a very weak emission. This suggests ACQ in **RhB-C18**.^11^ Furthermore, the addition of a nonionic surfactant (Triton X-100, 0.3 vol%) to the **RhB-C18** aqueous solution drastically increased the PL intensity (Fig. S2b†), which suggests the dissociation of the **RhB-C18** supramolecular assembly by Triton X-100. The photophysical properties of the **Rh19-C2/Rh19-C18** pair exhibited the same trend as those of the **RhB-C2/RhB-C18** pair (Fig. S1c, d and S2c, d†). **Rh19-C18** formed supramolecular assemblies, whereas **Rh19-C2** did not form supramolecular assemblies in water. With the increasing concentration, the absorbances of the rhodamine derivatives measured in water increased linearly (Fig. S3 and S4†); this indicates that **RhB-C2** and **Rh19-C2** did not form supramolecular assemblies until 50 μM (Fig. S3†), and the excessive aggregation of **RhB-C18** and **Rh19-C18** did not occur at least until 100 μM (Fig. S4†).

Dynamic light scattering (DLS) measurements and transmission electron microscopy (TEM) observations were performed to confirm the formation of supramolecular assemblies. DLS measurements indicated the presence of supramolecular assemblies of **RhB-C18** and **Rh19-C18** having average sizes of 200 and 82 nm, respectively (Fig. S5a and b†). TEM revealed the formation of spherical supramolecular assemblies of **RhB-C18** and **Rh19-C18** (Fig. S5c and d†). The selected area electron diffraction (SAED) patterns obtained by TEM exhibited diffused rings and no diffraction spots, which indicate the amorphous nature of the rhodamine supramolecular assemblies (Fig. S5e and f†).

### Photocatalytic activities of the rhodamines in water

**Fig. 3:**
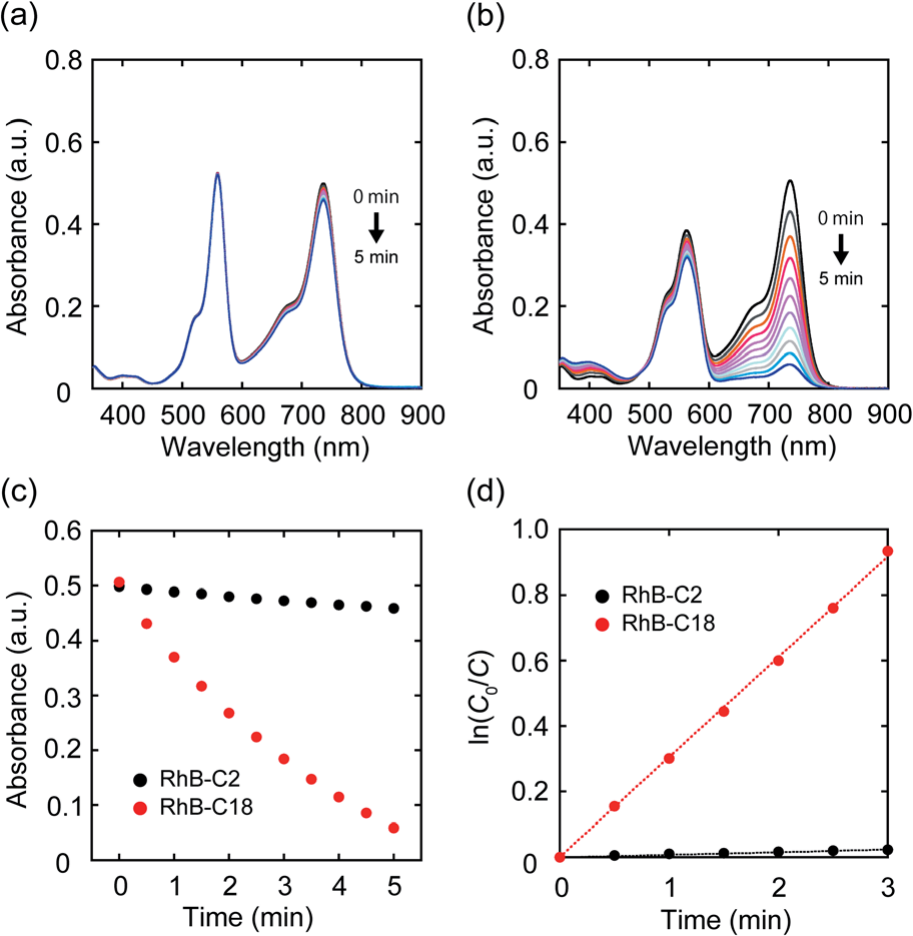
(a, b) UV-vis absorption spectra of the mixture of **HITCI** with (a) **RhB-C2** and (b) **RhB-C18** after photoirradiation. (c) Time-course of absorbance at 735 nm after photoirradiation using **RhB-C2** (black circle) and **RhB-C18** (red circle). (d) Linear regression plots of **HITCT** absorbance with **RhB-C2** (black circle) and **RhB-C18** (red circle). Experimental conditions: [**RhB-C2**] = [**RhB-C18**] = 5.0 μM, [**HITCI**] = 2.5 μM solvent: water, rt, irradiation wavelength: 560 nm (FWHM: 10 nm, Xe lamp, 300 W).

Since it became clear that the rhodamine pairs were suitable for evaluation of AI-PCA, we initially examined their photocatalytic activities in water using 1,1′,3,3,3′,3′-hexamethylindotricarbocyanine iodide (**HITCI**) (Fig. S6a†).^29^ The rhodamines and **HITCI** were mixed in water, and the UV-vis spectra were recorded after photoirradiation of the mixture (*λ* = 560 nm (FWHM: 10 nm, Xe lamp, 300 W)). In the case of **RhB-C2**, the absorption band at around 735 nm arising from **HITCI** gradually decreased (Fig. 3a). Considering the self-photooxidation of **HITCI** upon photoirradiation (Fig. S6b and c†), the photocatalytic activity of **RhB-C2** in **HITCI** oxidation is considered negligible. In contrast, **RhB-C18** remarkably oxidized **HITCI** within a short period of photoirradiation (Fig. 3b and c). In addition, similar to **RhB-C2**, **Rh19-C2** caused slow degradation of **HITCI** (Fig. S7a†), whereas the supramolecular assemblies of **Rh19-C18** rapidly degraded **HITCI** (Fig. S7b and c†). The rate of **HITCI** oxidation by the rhodamines was estimated to be 8.2 × 10^−3^, 3.1 × 10^−1^, 2.9 × 10^−3^, and 1.0 × 10^−1^ min^−1^ for **RhB-C2**, **RhB-C18**, **Rh19-C2**, and **Rh19-C18**, respectively, by linear regression fitting (Fig. 3d and S7d†). Compared with **RhB-C2** and **Rh19-C2**, **RhB-C18** and **Rh19-C18** accelerated **HITCI** oxidation 39-fold and 34-fold, respectively. Additionally, in the presence of Triton X-100, the rates of **HITCI** photooxidation by the **RhB-C2/RhB-C18** and **Rh19-C2/Rh19-C18** pairs were almost the same (Fig. S8†). These results indicate that the formation of supramolecular assemblies of rhodamines enhanced the oxidation of **HITCI**.

### Mechanistic study on the photocatalytic activity of the rhodamine SA-SPCs

**Fig. 4:**
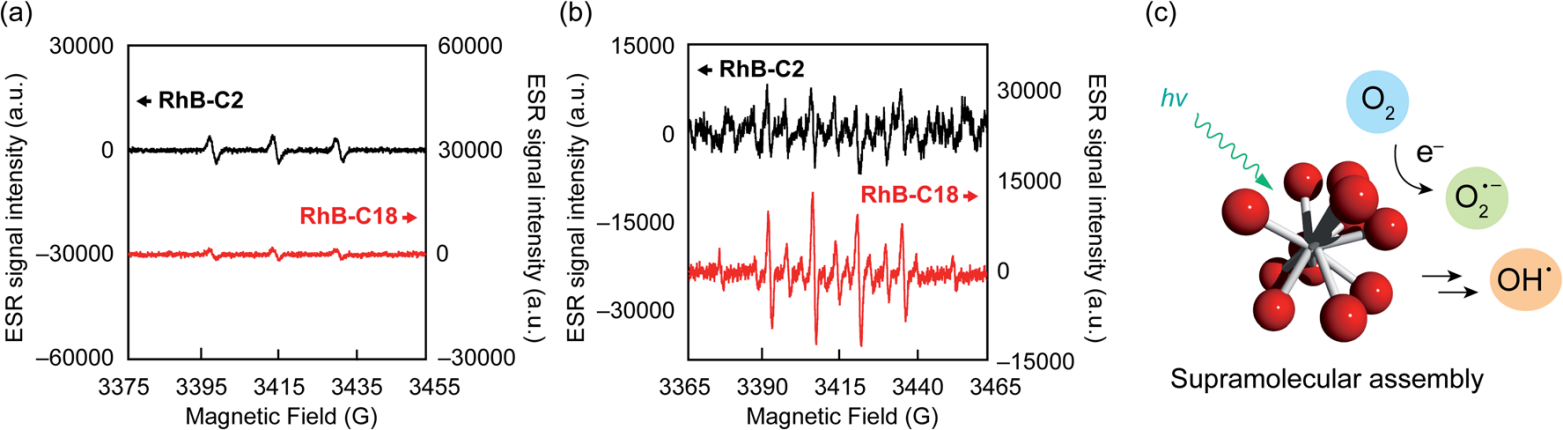
ESR signals of (a) 4-OH-TEMP and (b) DMPO adducts observed for **RhB-C2** and **RhB-C18** after light irradiation. (c) Schematic representation of generation of a hydroxyl radical through electron transfer and chain reactions. Experimental conditions: [**RhB-C2**] = [**RhB-C18**] = 50 μM, [**4-OH-TEMP**] = [**DMPO**] = 100 mM, solvent: water, rt, irradiation wavelength: 560 nm (FWHM: 10 nm, Xe lamp, 300 W, 3 min).

The photoreaction of SA-SPCs is considered to occur *via* two types of mechanisms involving electron and/or energy transfer processes.^30^ Under aerobic conditions, in the energy transfer process, singlet oxygen (^1^O_2_) is commonly involved in the photocatalytic reaction, whereas oxygen radicals such as superoxide anion radicals (O_2_˙^−^) and hydroxyl radicals (OH˙^−^) are involved in the electron transfer mechanism (Fig. S9†).^31^ To explain the mechanism of the photocatalytic reaction, electron spin resonance (ESR) spectroscopy was performed using 4-hydroxy-2,2,6,6-tetramethylpiperidine (4-OH-TEMP)^32^ and 5,5-dimethyl-1-pyrroline *N*-oxide (DMPO)^33^ as spin trap reagents to detect ^1^O_2_ and oxygen radicals (*e.g.* O_2_˙^−^, OH˙^−^), respectively (Fig. S10a and b†). The ESR experimental conditions were first determined using Rose Bengal (Fig. S10c†) as a standard.^34^ The ESR spectra of **RhB-C2** and **RhB-C18** exhibited a characteristic 1 : 1 : 1 triplet corresponding to the TEMPOL radical (Fig. 4a). **RhB** generates a triplet state despite the low quantum yield of intersystem crossing (quantum yield (*Φ*_T_): 0.006).^35^ Therefore, it is reasonable that **RhB-C2** exhibited an ESR signal for the TEMPOL radical. No significant differences were observed in the signal intensities of the TEMPOL radicals of **RhB-C2** and **RhB-C18**, which implies that the rate of energy transfer to oxygen did not drastically change after self-assembly. These results suggest that the photocatalytic reaction of **RhB-C18** does not occur through the energy transfer mechanism. Subsequently, we evaluated the generation of oxygen radical species *via* the electron transfer mechanism. The ESR spectrum for **RhB-C2** in DMPO did not exhibit a clear signal for a DMPO adduct (Fig. 4b), whereas the ESR spectrum of **RhB-C18** exhibited a signal for a DMPO hydroxyl radical adduct (DMPO-OH). Since the superoxide anion (O_2_˙^−^) is unstable in aqueous media, it reacts with protons immediately upon addition to an aqueous medium (Fig. S9a†) and does not react with DMPO to form DMPO-OOH. Hence, no DMPO-OOH peak appeared in the ESR spectrum. However, we confirmed the generation of a hydroxyl radical (OH˙^−^) that was produced by the chain reaction starting from O_2_˙^−^ ions through electron transfer in water (Fig. 4b, c and S9a†). The experimental results showed that **RhB-C18** exhibits photocatalytic activity mainly through an electron transfer mechanism. Further, the ESR spectra of the **Rh19-C2/Rh19-C18** pair were similar to those of the **RhB-C2/RhB-C18** pair (Fig. S11†).

### Photocatalytic hydrogen evolution using the rhodamine SA-SPCs

**Fig. 5:**
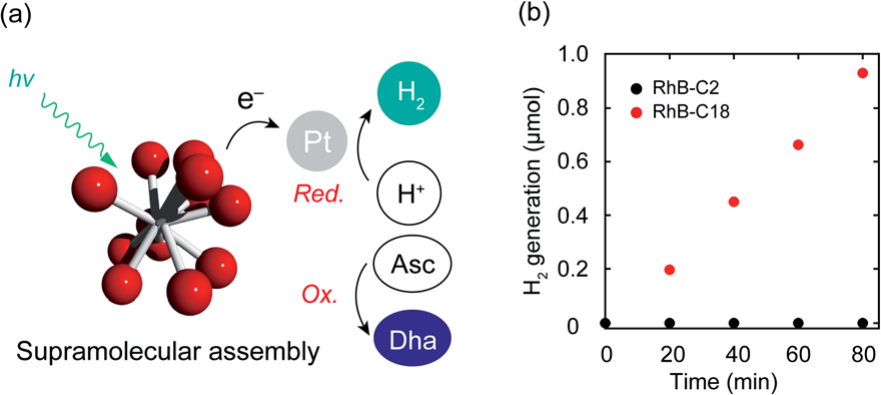
(a) Schematic representation of photocatalytic hydrogen evolution by the SA-SPC composed of rhodamine derivatives. (b) Time-course of hydrogen evolution by **RhB-C18** and **RhB-C18** under visible light. Experimental conditions: [rhodamines] = 50 μM, [Pt] = 100 μM, [Asc] = 500 mM, solvent: water, rt, irradiation light wavelength: >360 nm. Asc: ascorbic acid, DHA: dehydroascorbic acid.

Photocatalytic hydrogen evolution is one of the important reactions toward realizing artificial photosynthesis.^2^ The hydrogen evolution abilities of various photocatalysts have been actively examined to date. The electron transfer mechanism of the SA-SPC encouraged us to investigate the photocatalytic hydrogen evolution by the rhodamine SA-SPCs using Pt nanoparticles as a co-catalyst and ascorbic acid (Asc) as a sacrificial reagent (Fig. 5a). The HOMO levels of the rhodamine derivatives (Table S1,†**RhB-C2**: −5.4 eV, **RhB-C18**: −5.5 eV **Rh19-C2**: −5.4 eV, and **Rh19-C18**: −5.5 eV) determined by cyclic voltammetry or square wave voltammetry (Fig. S12 and S13†) were sufficiently lower than that of Asc (−4.6 eV),^36^ while the LUMO levels of the rhodamine derivatives (Table S1,†**RhB-C2**: −3.2 eV, **RhB-C18**: −3.5 eV **Rh19-C2**: −3.1 eV, and **Rh19-C18**: −3.4 eV) were enough for the proton reduction reaction. The addition of Asc and H_2_PtCl_6_ as a precursor of Pt nanoparticles hardly affected the size of SA-SPCs (Fig. S14a and b†).

The time-courses of photocatalytic hydrogen evolution by **RhB-C2** and **RhB-C18** are shown in Fig. 5b. After light irradiation (*λ* > 360 nm, 300 W (Xe lamp)), Pt nanoparticles were formed (Fig. S14c–f†) and **RhB-C18** exhibited hydrogen generation, while **RhB-C2** did not display hydrogen evolution. The **RhB-C18** SA-SPC functioned for 80 min without any decrease in the photocatalytic activity, and the average hydrogen evolution rate (HER) was determined to be 3.7 mmol g^−1^ h^−1^ (Fig. 5b), which is comparable to that of other excellent organic systems such as g-C_3_N_4_ (0.67 mmol g^−1^ h^−1^)^37^ and a covalent organic framework (10.1 mmol g^−1^ h^−1^).^38^ 80 min after light irradiation, the photocatalytic activities of the SA-SPCs decreased due to the decomposition of rhodamines. **Rh19-C18** exhibited photocatalytic hydrogen evolution (HER: 2.9 mmol g^−1^ h^−1^), while **Rh19-C2** did not (Fig. S15a†). One of the reasons for the high HER would be intermolecular electron migration among the rhodamines.^39^ The photoirradiation of rhodamines generated intermolecular charge separation states, and the migration between the rhodamines may have facilitated efficient electron transfer to the Pt nanoparticle. The apparent quantum efficiencies of **RhB-C18** and **Rh19-C18** were 0.059 and 0.039% under these conditions, respectively.

To examine the effects of the SA-SPC concentration on the hydrogen evolution reaction, the SA-SPC concentrations were increased from 50 to 100 μM. The amorphous self-assembled spherical structures were almost unchanged (Fig. S16,† average particle diameter: **RhB-C18**: 223 nm, **Rh19-C18**: 122 nm) after the increase. The hydrogen evolution rates decreased from 3.7 to 2.4 mmol g^−1^ h^−1^ for **RhB-C18** (Fig. S17a†). On the other hand, in the case of **Rh19-C18**, the hydrogen evolution rate significantly increased from 2.9 to 5.9 mmol g^−1^ h^−1^ (Fig. S17b†). These results indicate that the hydrogen evolution rates are significantly affected by the concentration of the SA-SPC. The versatile factors including the size, morphology, surface area, fluidity, and electric state of the SA-SPC, and interactions between the SA-SPC and Pt nanoparticles or ascorbic acid would have a sensitive effect on the hydrogen evolution. A detailed understanding of the changes of **RhB-C18** and **Rh19-C18** for photocatalytic hydrogen evolution rates is currently difficult. We will study how each factor has effects on photocatalytic activity in future studies.

## Conclusions

In summary, we demonstrated the AI-PCA of two rhodamine derivatives. The rhodamine SA-SPCs showed excellent photocatalytic hydrogen evolution rates (up to 5.9 mmol g^−1^ h^−1^). ESR spectroscopic analysis revealed that the photocatalytic reaction proceeded *via* an electron transfer mechanism. We think that the concept of AI-PCA might be applicable to a wide range of photoactive molecules. Further investigations on the effects of organic dyes, morphologies, and the molecular arrangements of supramolecular assemblies on the photocatalytic activity of SA-SPCs, and the detailed mechanism of AI-PCA are currently underway.

## Conflicts of interest

There are no conflicts to declare.

## Supplementary Material

SC-011-D0SC04285D-s001
