# Supplementary material for: Aggregation-induced photocatalytic activity and efficient photocatalytic hydrogen evolution of amphiphilic rhodamines in water
Source: Chem Sci. 2020 Oct 7;11(43):11843–8. doi: 10.1039/d0sc04285d (PMC8162825; doi:10.1039/d0sc04285d)
Supplement: SC-011-D0SC04285D-s001 [file SC-011-D0SC04285D-s001.pdf]

Electronic Supplementary Material (ESI) for Chemical Science.
This journal is © The Royal Society of Chemistry 2020

Supporting Information

Aggregation-induced photocatalytic activity and efficient photocatalytic hydrogen evolution of amphiphilic rhodamines in water

Hajime Shigemitsu,[a,b,c] Youhei Tani,[a] Tomoe Tamemoto,[a] Tadashi Mori,[a] Xinxi Li,[d] Yasuko Osakada,[d,e] Mamoru Fujitsuka,[d] Toshiyuki Kida[a]

[a]Department of Applied Chemistry, Graduate School of Engineering, Osaka University, Suita 565-0871, Japan

[b]Frontier Research Base for Global Young Researchers, Graduate School of Engineering, Osaka University, Suita 565-0871, Japan

[c]Global Center for Medical Engineering and Informatics, Osaka University, Suita 565-0871, Japan

[d]Institute of Scientific and Industrial Research (SANKEN), Osaka University, Mihogaoka 8-1, Ibaraki, Osaka 567-0047, Japan

[e]Institute for Advanced Co-creation Studies, Osaka University, 1-1 Yamadagaoka, Suita, Osaka, 565-0871, Japan

Table of contents

1. Materials, Instruments, and Methods S2

2. Synthetic Methods S5

3. Supplementary Figures S7

1. Materials, Instruments, and Methods

Materials and Instruments

Unless stated otherwise, all commercial reagents were used as received. **RhB-C18** and **Rh19-C2** were purchased from Tokyo Chemical Industry (TCI) (**RhB-C18**: Octadecyl rhodamine B chloride (counter anion: chloride ion, product number: O0512), **Rh19-C2**: Rhodamine 6G (counter anion: chloride ion, product number: R0039)). Water was purified using a Milli-Q water system (Direct-Q 3UV, Millipore) ^{1}H and ^{13}C NMR spectra were recorded on a JEOL JNM-ESC400. Data are reported as follows: chemical shifts in ppm using the residual solvent peak (CD_3OD: 3.31 ppm) as an internal standard, integration, multiplicity (s = singlet, d = doublet, t = triplet, m = multiplet, br = broadened), and coupling constants (Hz). High-resolution mass spectra were recorded on a JEOL JMS-700. UV-vis absorption and photoluminescence spectra were recorded on a JASCO V-660 and JASCO FP-8500, respectively. Dynamic light scattering (DLS) measurements were conducted by Malvern Panalytical Zetasizer Nano ZS. For TEM analysis, samples were deposited onto formvar support film-coated Cu grids that were subjected to hydrophilic treatment for 60 s using a JEOL HDT-400 device. To examine the photocatalytic activities of the samples toward **HITCI** (Sigma-Aldrich, product number: 252034-100MG) and for ESR spectroscopy experiments, the samples were irradiated with an ASAHI SPECTRA MAX-301 Xe light source (300 W) with a light band-pass filter (560 nm (FWHM: 10 nm). Electron spin resonance (ESR) spectroscopy was performed using 4-OH-TEMP (TCI, product number: T0910) and DMPO (TCI, product number: D2362) as spin-trapping reagents for the detection of singlet oxygen and hydroxyl radical species, respectively, on a BRUKER EMXmicro spectrometer. Cyclic voltammetry and square wave voltammetry were performed on an electrochemical workstation (ALS600B, BAS) using a conventional three-electrode configuration. The device includes a diamond working electrode, a Pt wire counter electrode, and a Ag/AgCl reference electrode. The electrolyte was an argon-purged water solution containing 0.1 M KCl. The amount of generated H_2 was detected using a gas chromatograph equipped with a TCD detector (GC-8A, Shimadzu, Kyoto, Japan). For the photocatalytic hydrogen evolution experiments, irradiation was performed with a ASAHI SPECTRA HAL-320W Xe lamp (300 W) with a light cut-off filter (<360 nm).

UV-vis and PL spectroscopies

The solution state UV-vis absorption and photoluminescence spectra were measured on a V-660 spectrometer (JASCO, Tokyo, Japan) and FP-8500 fluorescence spectrometer (JASCO, Tokyo, Japan) using 1.0 × 1.0 cm^2 or 0.2 × 1.0 cm^2 cuvette, respectively. The samples were prepared by diluting a 10 mM DMSO stock solution with DMSO or water. The DMSO contents of all the samples were kept below 0.1 vol% without the experiments using DMSO as a solvent. The spectra were recorded within 3 h of sample preparation at room temperature. The excitation wavelengths used in PL spectroscopy are mentioned in the figure captions.

Dynamic light scattering (DLS) experiments

The sample solutions used for DLS measurements were prepared by the same method as that used for preparing the samples for UV-vis and PL spectroscopies. The sample solutions were then filtered through a PTFE membrane filter (pore size: 200 nm, ADVANTEC DISMIC-25JP) to remove contaminants that may hinder DLS measurements. The samples were poured into plastic cuvettes and DLS measurements were made at room temperature at a scattering angle of 90° and a laser wavelength of 633 nm.

Transmission electron microscopy (TEM) observations

The hydrophilic treatment of the formvar support films coated Cu grid (Cu 200 mesh covered with a formvar membrane, JEOL) were performed by JEOL HDT-400 for 60 sec. The sample solutions (50 μM, 5.0 μL) were dropped on the hydrophilic treated formvar membrane coated Cu grid. The samples were dried at room temperature for 10 min and then in *vacuo* over 12 h. The obtained samples are observed by JEOL ARM200F at an accelerator potential of 200 kV.

Examination of photocatalytic activity of rhodamine derivatives toward HITCI

A **HITCI** DMSO stock solution (500 μM, 3.0 μL) was added to the prepared sample solutions (3.0 mL) in 1.0 × 1.0 cm^2 cuvettes. The solution mixtures were then irradiated using a Xe lamp with a band-pass filter (560 nm (FWHM: 10 nm)) with continuous stirring to initiate the photoreaction. To monitor the course of the reaction, UV-vis spectra were recorded every 1 min for 5 min.

ESR experiments using spin trap reagents

The prepared sample solutions ([**RhB-C2**] = [**RhB-C18**] = [**Rh19-C2**] = [**Rh19-C18**] = 50 μM, [4-OH-TEMP] = [DMPO] = 100 mM) were poured into quartz capillary tubes (φ: 2 mm, kk-agri) and irradiated with a Xe lamp with a band-pass filter (560 nm (FWHM: 10 nm)) (singlet oxygen detection (4-OH-TEMP); 3 min, hydroxy radical detection (DMPO): 10 min). The distance between the sample and the light source was 2.0 cm. Immediately after photoirradiation, the ESR spectra were recorded with a center field of 3415 G, sweep width of 100 G, and microwave power of 1.0 mW at room temperature.

Redox potential

Cyclic voltammetry and square wave voltammetry were performed using a three-electrode setup in a 0.1 M KCl aqueous solution ([Rhodamine derivatives] = 100 μM) in the potential range of 0.2–1.2 V at 50 mV/s using a diamond working electrode (HOKUTO DENKO, HX-W7), a platinum wire counter electrode, and a Ag/AgCl reference electrode. The HOMO levels were estimated by converting the observed peak potential to standard calomel electrode (SCE) by subtracting 0.042 V,

and then subtracting the potential from –4.44 eV. The LUMO was estimated by converting the absorption edge wavelength to optical band gap energy (eV) and adding the value to the calculated HOMO level.

Hydrogen evolution experiments

A test tube was charged with an aqueous solution of the supramolecular photocatalysts or monomers of rhodamine (50 or 100 μM), an aqueous solution of ascorbic acid (500 mM), and hexachloroplatinic acid (50 μM) as a Pt nanoparticle precursor, and the resulting solution was kept at room temperature for 15 min. The test tube was sealed with a W-18 rubber cap (Sansho, Osaka, Japan) and the mixture was degassed by bubbling argon for 30 min. The solution was then irradiated with a Xe light source (300 W) for 80 min. Subsequently, gas samples were taken with a gas-tight syringe and analyzed using a gas chromatograph equipped with a TCD detector (GC-8A, Shimadzu, Kyoto, Japan). The column temperature was 100 °C, the detector current was 60 mA, the primary pressure was 600 kPa, and the carrier gas pressure was 300 kPa. A calibration curve was constructed using H_2 standard gas (1020–11201, GL science) to determine the amount of H_2. The apparent quantum efficiency (AQE) for the photocatalytic H_2 evolution was measured using a longpass filter (525 nm). The AQE were estimated using the equation below:

$$\text{AQE (\%)} = \frac{2 \times \text{the number of evolved hydrogen molecules}}{\text{the number of incident photons}} \times 100$$

2. Synthetic Methods

Synthesis of RhB-C2

Aqueous cesium carbonate (2 *N*, 1.0 mL) was added to a methanolic solution (6.0 mL) of rhodamine B (240 mg, 0.53 mmol), and the solution mixture was boiled, cooled to 4 °C, and maintained at this temperature for 12 h. The generated precipitate was filtered, washed with cold methanol, and dried under vacuum at 60 °C to obtain a Cs salt of rhodamine B as a dark violet solid (238 mg, 0.435 mmol, 82%). This material was used in the next step without further purification.

A mixture of Cs-salt of rhodamine B (70 mg, 0.13 mmol) and 1-iodoethane (47 μL, 0.192 mmol) in DMF (1.5 mL) was stirred at 60 °C for 19 h. The solvent was removed under reduced pressure, and the residue was purified by column chromatography on silica gel with a mixture of chloroform: methanol (9:1, v/v) as eluent to afford **RhB-C2** (counter anion: iodide ion) as a dark purple solid (70 mg, 0.12 mmol, 94 %).

^{1}H NMR (400 MHz, CD_3OD, rt): δ (ppm) = 8.31 (dd, *J* = 7.6, 1.2 Hz, 1H), 7.84 (m, 2H), 7.44 (dd, *J* = 7.6, 1.2 Hz, 1H), 7.13 (d, *J* = 9.2 Hz, 2H), 7.04 (dd, *J* = 9.2, 2.4 Hz, 2H), 6.99 (d, *J* = 2.4 Hz, 2H), 4.02 (q, *J* = 7.2 Hz, 2H), 3.67 (q, *J* = 7.2 Hz, 8H), 1.31 (t, *J* = 7.2 Hz, 12H), 0.98 (t, *J* = 7.2 Hz, 3H). ^{13}C NMR (400 MHz, CD_3OD, rt): δ (ppm) = 166.6, 160.3, 159.3, 157.2, 134.0, 132.4, 132.2, 131.6, 131.5, 115.5, 114.8, 97.2, 62.5, 46.8, 14.0, 12.8. HR-MS (FAB): Calcd. for $[M+H]^+$: m/z = 471.2642; Found: 471.2655.

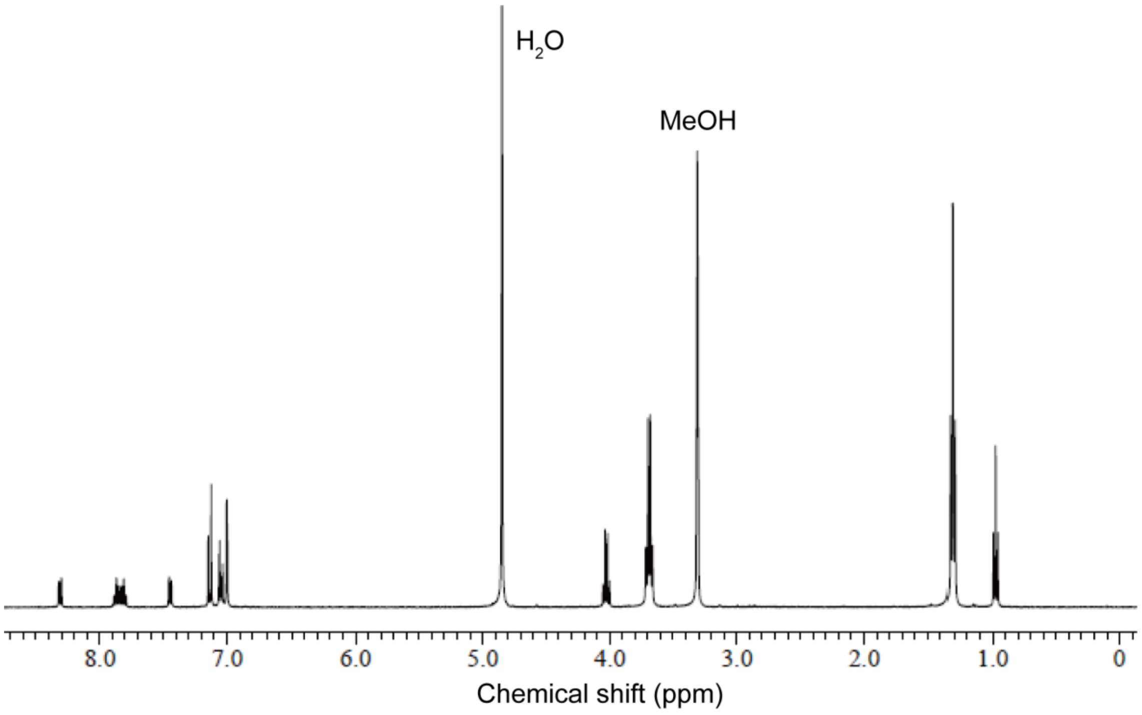

^{1}H NMR spectrum of **RhB-C2** (400 MHz, CD_3OD, rt).

Synthesis of Rh19-C18

An aqueous solution of cesium carbonate (2 *N*, 1.0 mL) was added to a methanolic solution (6.0 mL) of rhodamine 19 perchlorate (514 mg, 1.00 mmol), and the solution mixture was boiled, cooled to 4 °C, and maintained at this temperature for 12 h. The generated precipitate was filtered, washed with cold methanol, and dried under reduced pressure at 60 °C to obtain a Cs salt of rhodamine 19 as a dark violet solid (602 mg, 0.93 mmol, 93%). This material was used in the next step without further purification.

A mixture of Cs-salt of rhodamine 19 (300 mg, 0.55 mmol) and 1-bromooctadecane (367 mg, 1.10 mmol) in DMF (10 mL) was stirred at 60 °C for 19 h. The solvent was removed under reduced pressure, and the residue was purified by column chromatography on silica gel with a mixture of chloroform: methanol (9:1, v/v) as eluent to afford **Rh19-C18** (counter anion: bromide ion) as a dark purple solid (117 mg, 0.18 mmol, 32 %).

^{1}H NMR (400 MHz, CD_3OD, rt): δ (ppm) = 8.31 (d, *J* = 7.6, 1.2 Hz, 1H), 7.84 (m, 2H), 7.41 (dd, *J* = 7.6, 1.2 Hz, 1H), 6.96 (s, 2H), 6.90 (d, *J* = 1.2 Hz, 2H), 3.91 (t, *J* = 6.4 Hz, 2H), 3.56 (q, *J* = 7.2 Hz, 4H), 2.14 (s, 6H), 1.38 (t, *J* = 7.2 Hz, 6H), 1.01–1.33 (m, 30H), 0.82–0.93 (m, 5H). ^{13}C NMR (400 MHz, CD_3OD, rt): δ (ppm) = 167.2, 159.6, 159.2 158.0, 135.0, 134.2, 132.5, 132.2, 131.8, 131.7, 130.3, 127.2, 115.0, 95.1, 66.9, 39.7, 33.3, 31.0, 30.7, 30.5, 29.6, 27.2, 23.9, 17.8, 14.6, 14.3. HR-MS (FAB): Calcd. for $[M+H]^+$: m/z = 667.4833; Found: 667.4835.

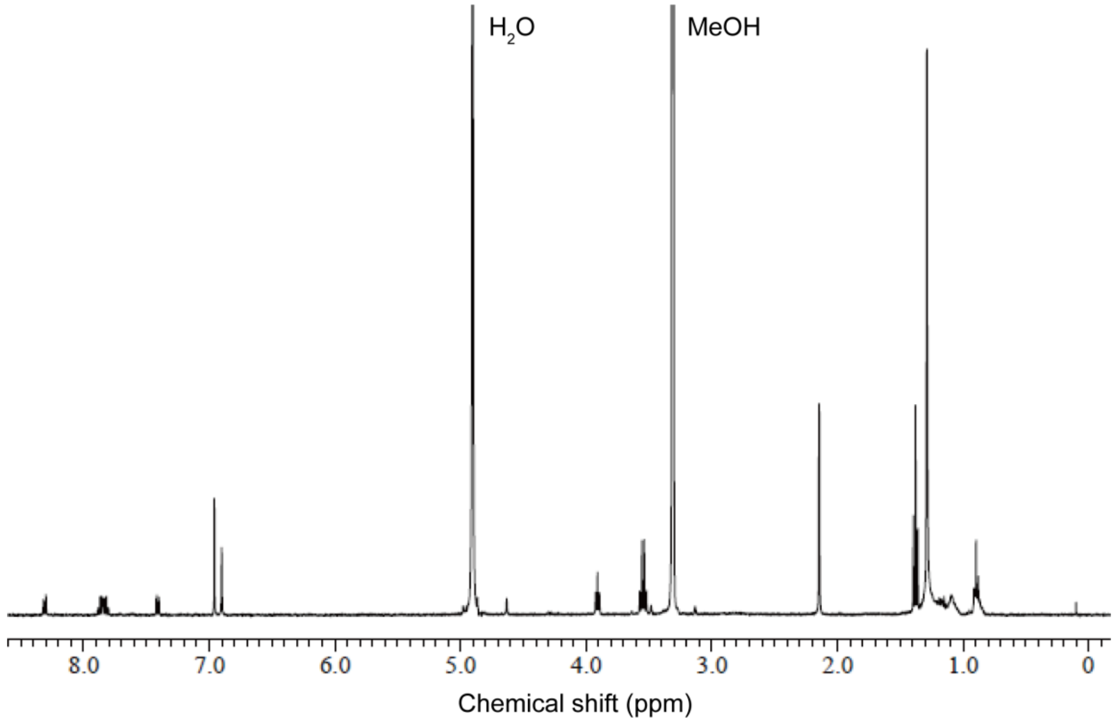

^{1}H NMR spectrum of **Rh19-C18** (400 MHz, CD_3OD, rt).

3. Supplementary Figures

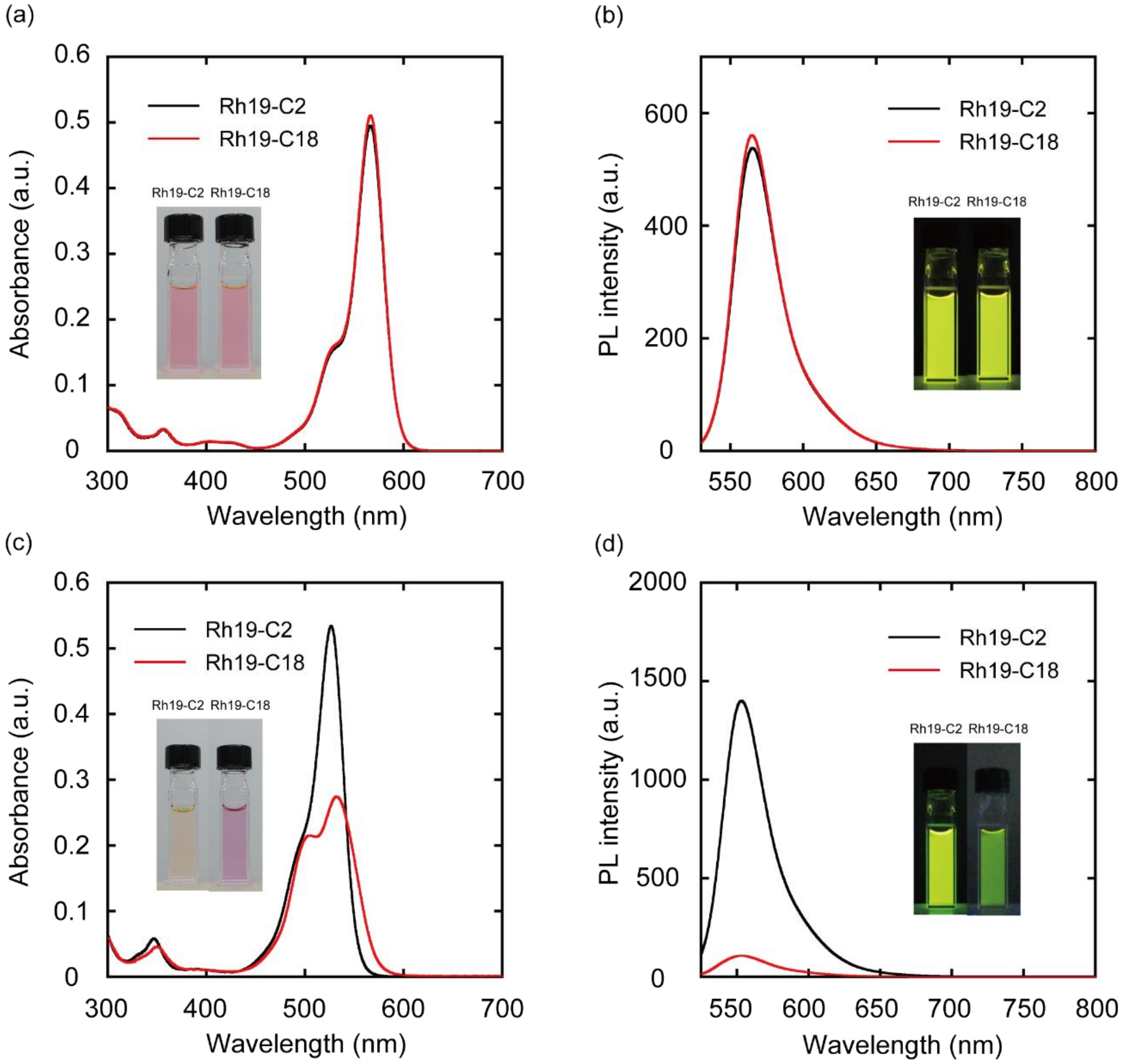

Figure S1. (a,c) UV-vis absorption and (b,d) PL spectra of **Rh19-C2** and **Rh19-C18** in (a,b) DMSO and (c,d) water. Experimental conditions: [**Rh19-C2**] = [**Rh19-C18**] = 5.0 μM, rt, water, excitation wavelength: 520 nm. Insets: Optical photos of **Rh19-C2** and **Rh19-C18** in DMSO and water under (a,c) daylight and (b,d) UV light (365 nm).

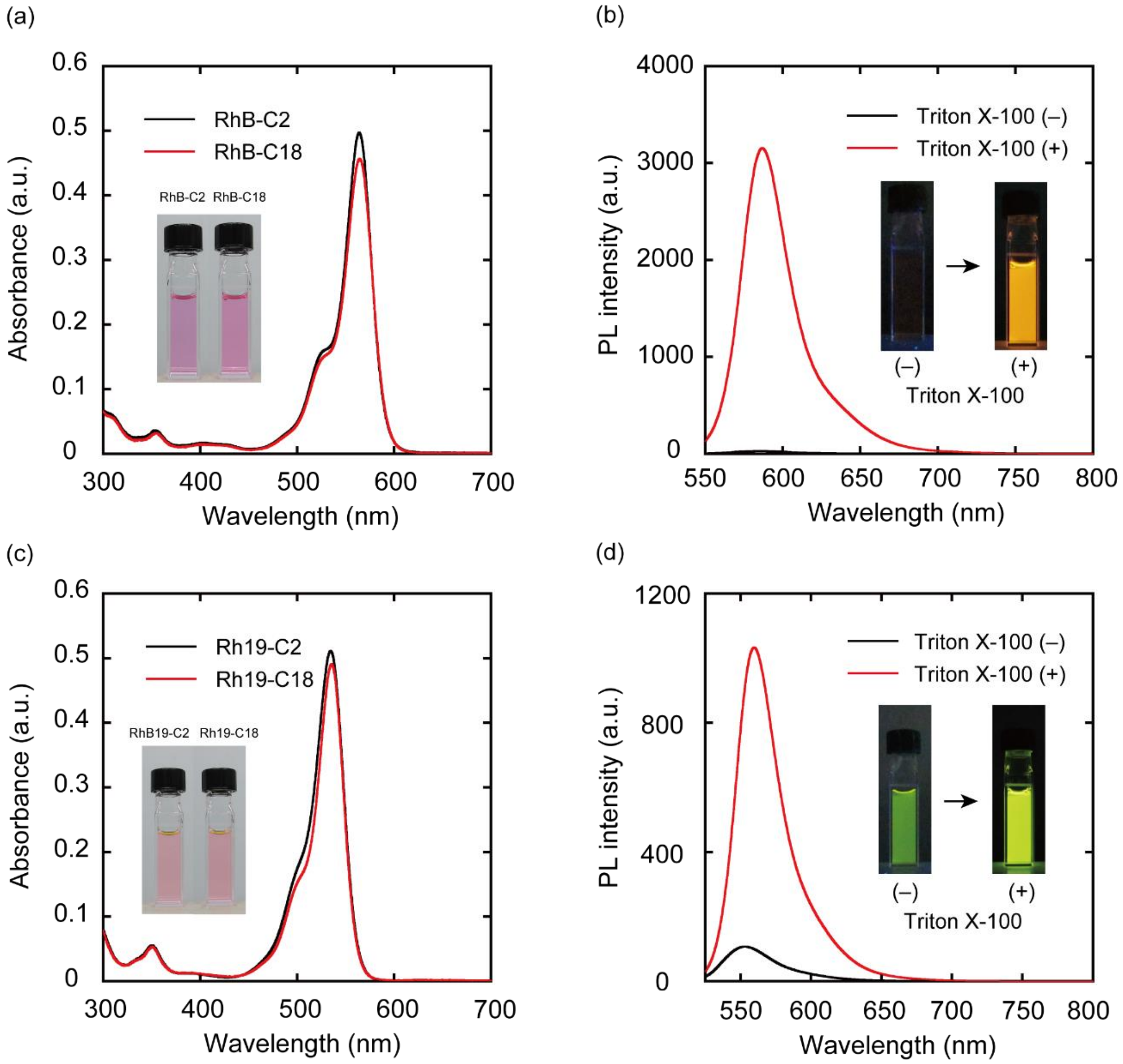

Figure S2. (a,c) UV-vis spectra of (a) **RhB-C2**/**RhB-C18** and (c) **Rh19-C2**/**Rh19-C18** in the presence of Triton X-100. (b,d) PL spectra of (b) **RhB-C18** and (d) **Rh19-C18** in water with or without Triton X-100. Experimental conditions: [**RhB-C2**] = [**RhB-C18**] = [**Rh19-C2**] = [**Rh19-C18**] =5.0 μM, [Triton X-100] = 0.3 vol%, rt, water, excitation wavelength: 540 (**RhB**) and 520 (**Rh19**) nm. Insets: Optical photos of (a,c) **RhB-C2**/**RhB-C18** and **Rh19-C2**/**Rh19-C18** in water under daylight, and (b,d) **RhB-C18** and **Rh19-C18** with or without Triton X-100 in water under UV light (365 nm).

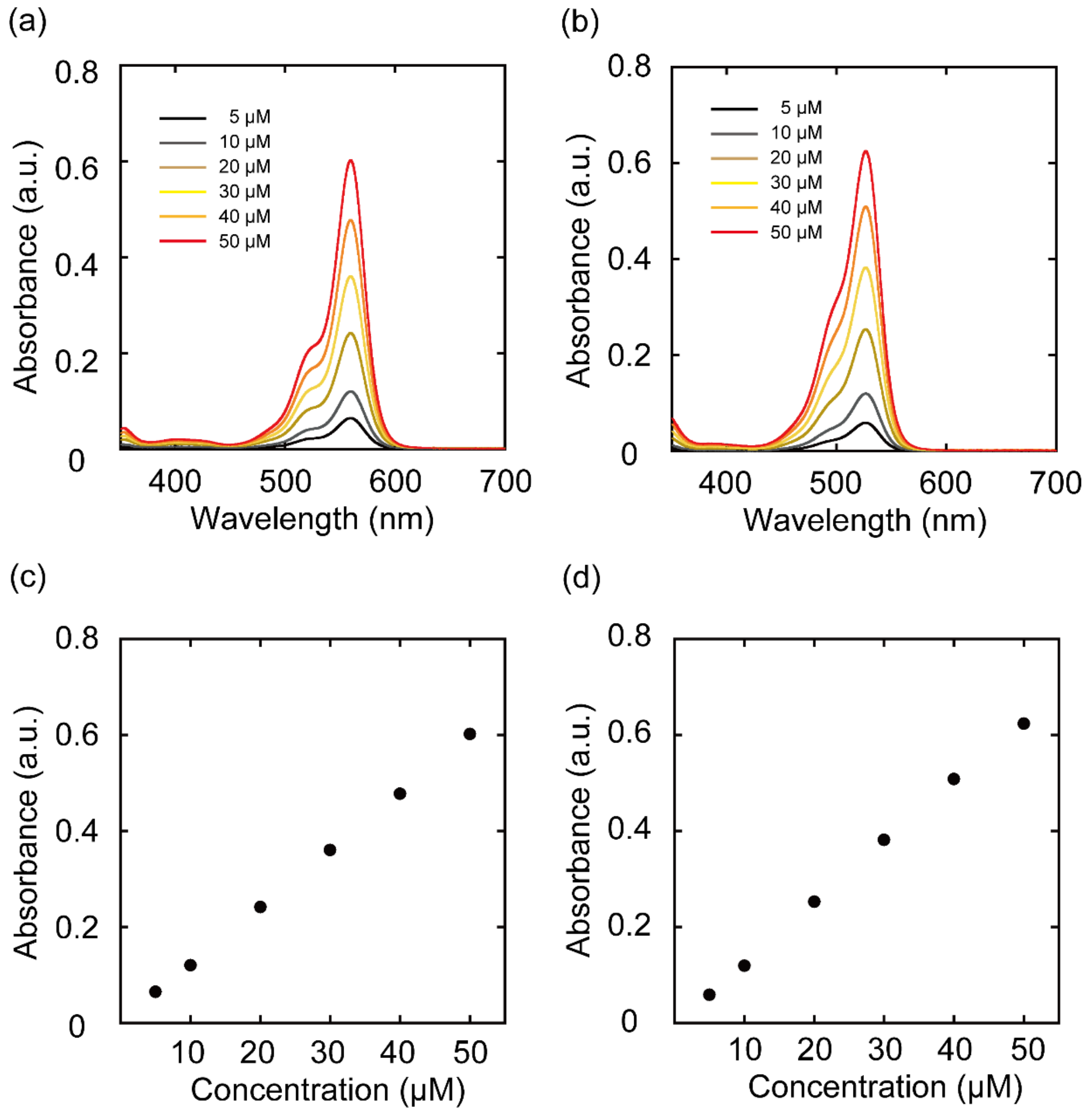

Figure S3. (a,b) Concentration-dependent UV-vis spectra of (a) **RhB-C2** and (b) **Rh19-C2**. (c,d) Variation in absorbance with rhodamine concentration: (c) **RhB-C2**, and (d) **Rh19-C2**. Experimental conditions: [**RhB-C2**] = [**Rh19-C2**] = 5.0–50 μM, rt, water. Optical path length: 0.2 mm.

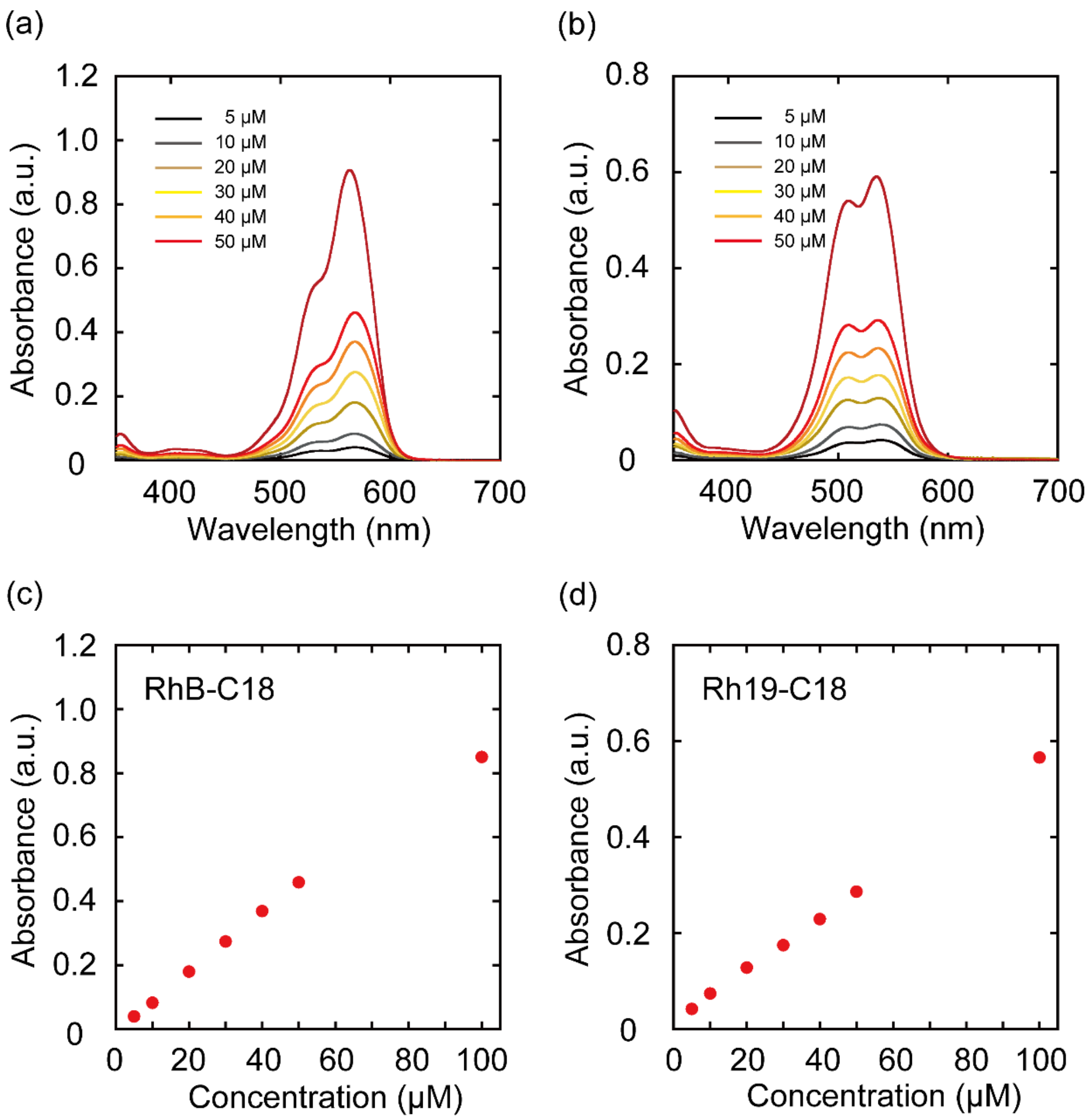

Figure S4. (a,b) Concentration-dependent UV-vis spectra of (a) **RhB-C18** and (b) **Rh19-C18**. (c,d) Variation in absorbance with rhodamine concentration: (c) **RhB-C18**, and (d) **Rh19-C18**. Experimental conditions: [**RhB-C18**] = [**Rh19-C18**] = 5.0–100 μM, rt, water. Optical path length: 0.2 mm.

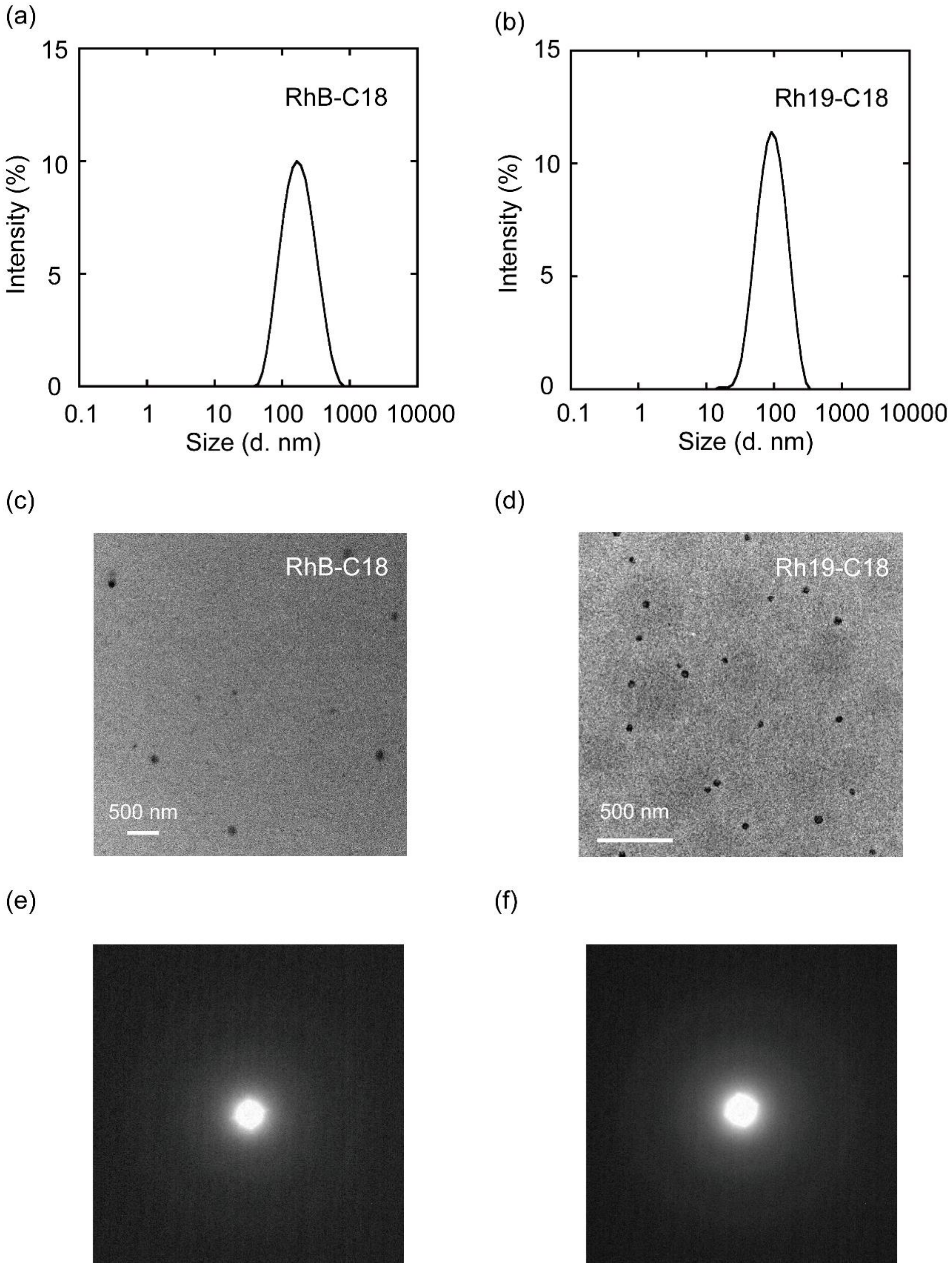

Figure S5. (a,b) Size distributions of supramolecular assemblies of (a) **RhB-C18** and (b) **Rh19-C18** determined by DLS (average particle size: (a) 200 nm, (b) 82 nm, Polydispersity index (PDI): (a) 0.410, (b) 0.182). (c,d) TEM images of supramolecular assemblies of (c) **RhB-C18** and (d) **Rh19-C18**. Scale bar: 500 nm. (e,f) SAED diffraction patterns of supramolecular assemblies of (e) **RhB-C18** and (f) **Rh19-C18**. Experimental conditions: [**RhB-C18**] = [**Rh19-C18**] = 50 μM, rt, water.

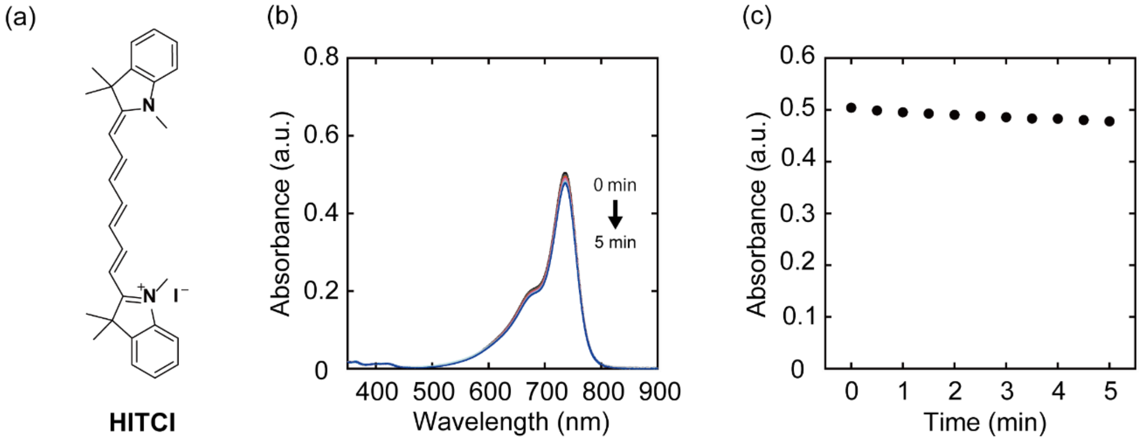

Figure S6. (a) Chemical structure 1,1',3,3,3',3'-hexamethylindotricarbocyanine iodide (**HITCI**). (b) UV-vis absorption spectra of **HITCI** after photoirradiation. (c) Time-course of absorbance at 735 nm after photoirradiation. Experimental conditions: [**HITCI**] = 2.5 μM solvent: water, rt, irradiation wavelength: 560 nm (FWHM: 10 nm, Xe lamp, 300 W).

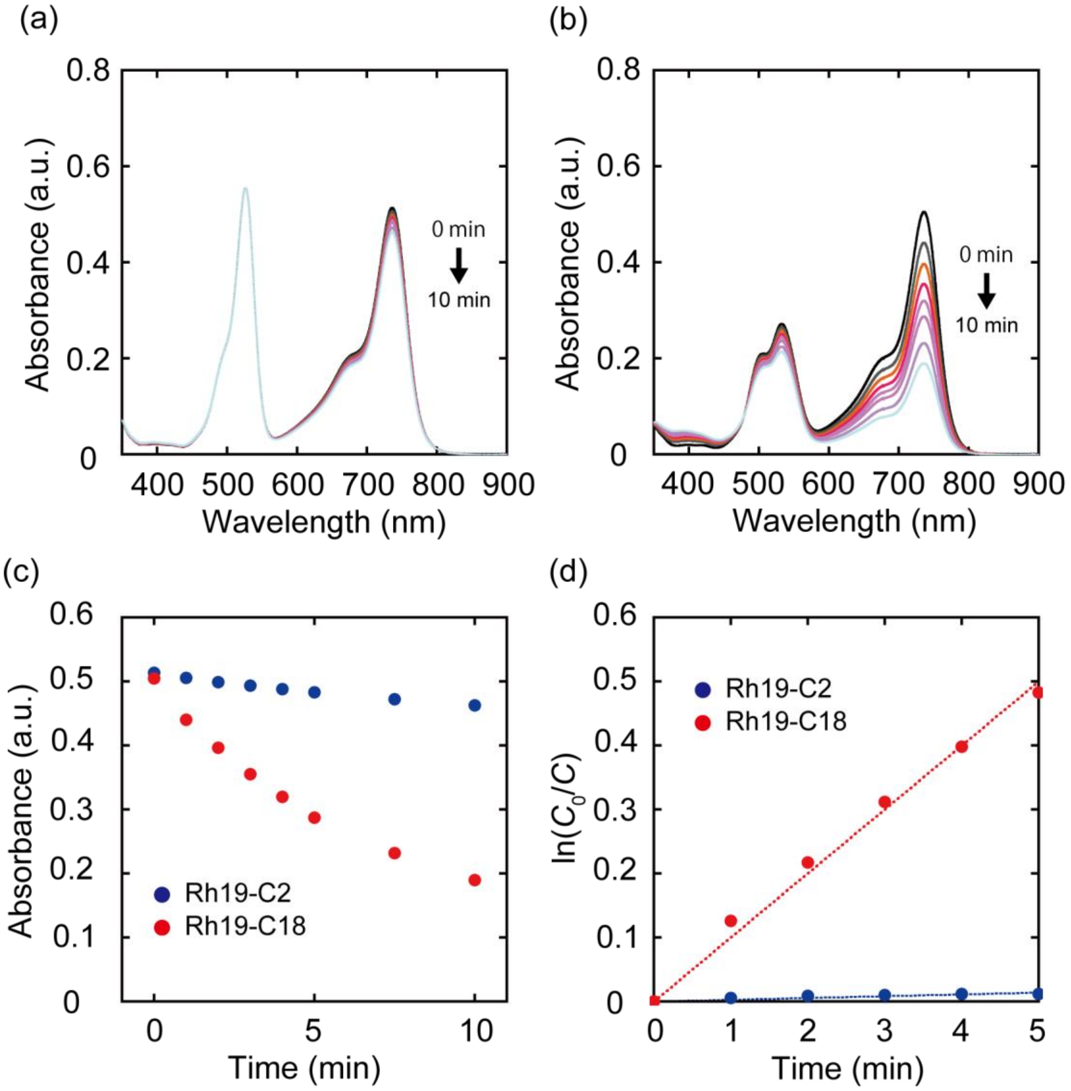

Figure S7. (a,b) UV-vis absorption spectra of mixture of **HITCI** with (a) **Rh19-C2** and (b) **Rh19-C18** after photoirradiation. (c) Time-course of absorbance at 735 nm after photoirradiation of samples containing **Rh19-C2** (blue circle) and **Rh19-C18** (red circle). (d) Linear regression plots of **HITCT** absorbance with **RhB-C2** (blue circle) and **RhB-C18** (red circle). Experimental conditions: [**RhB-C2**] = [**RhB-C18**] = 5.0 μM, [**HITCI**] = 2.5 μM solvent: water, rt, irradiation wavelength: 560 nm (FWHM: 10 nm, Xe lamp, 300 W).

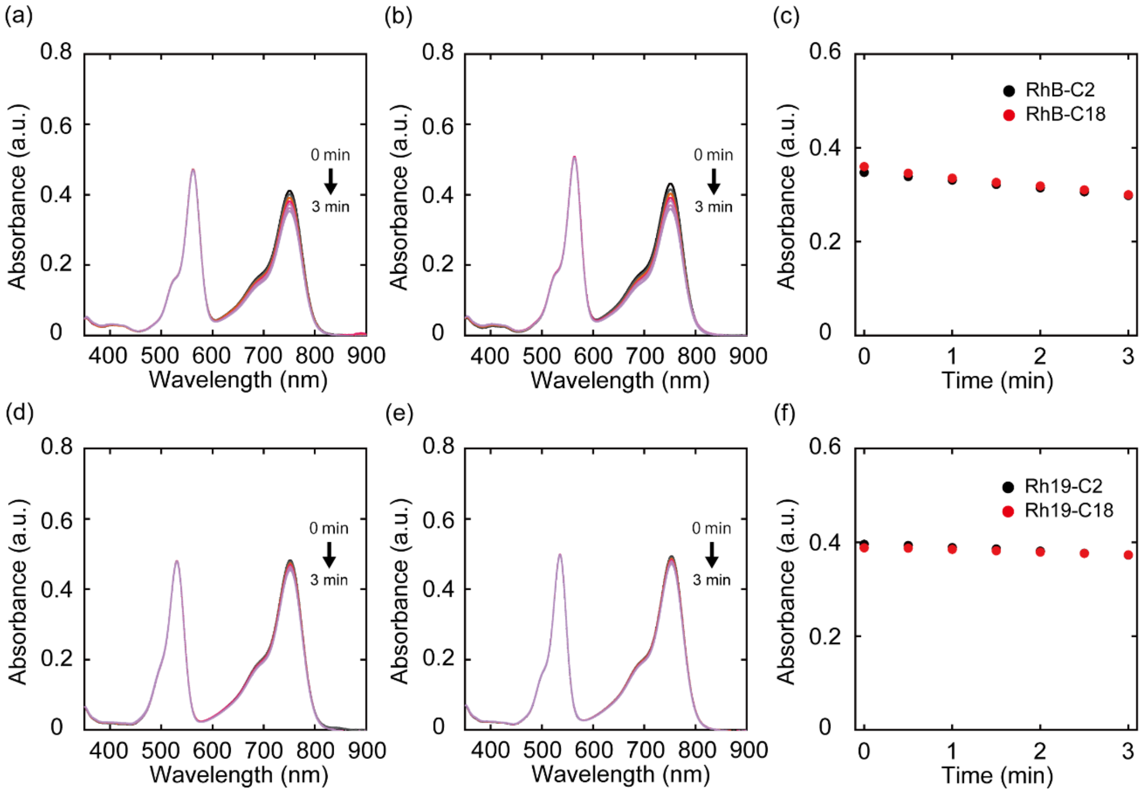

Figure S8. (a,b,d,e) UV-vis absorption spectra of mixtures of **HITCI** with (a) **RhB-C2**, (b) **RhB-C18**, (d) **Rh19-C2**, and (e) **Rh19-C18** in the presence of Triton X-100. (c,f) Time-course of absorbance at 735 nm after photoirradiation of a mixture of **HITCI** with (c) **RhB-C2/RhB-C18** and (f) **Rh19-C2/Rh19-C18** in the presence of Triton X-100.

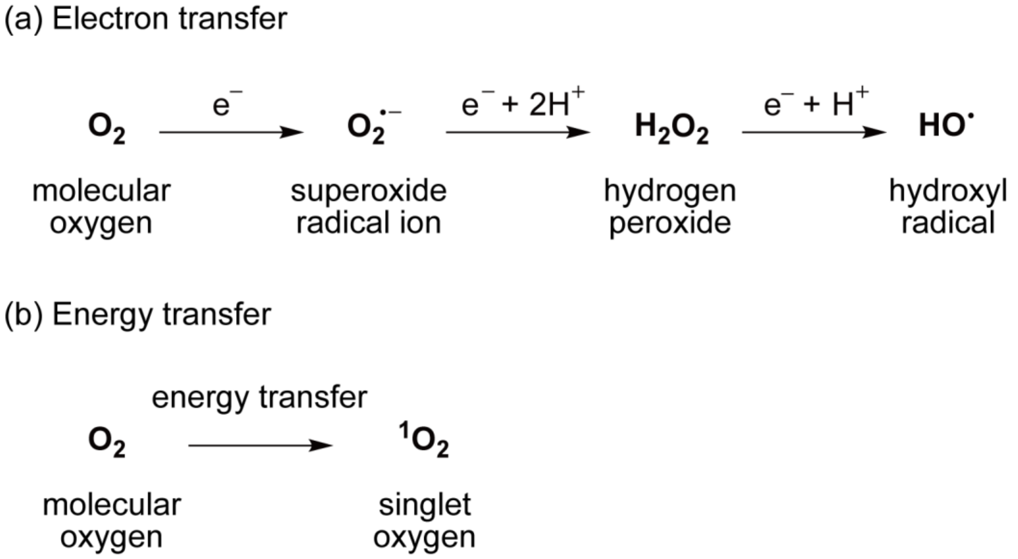

Figure S9. Formation of reactive oxygen species (ROS) through (a) electron- and (b) energy-transfer processes.

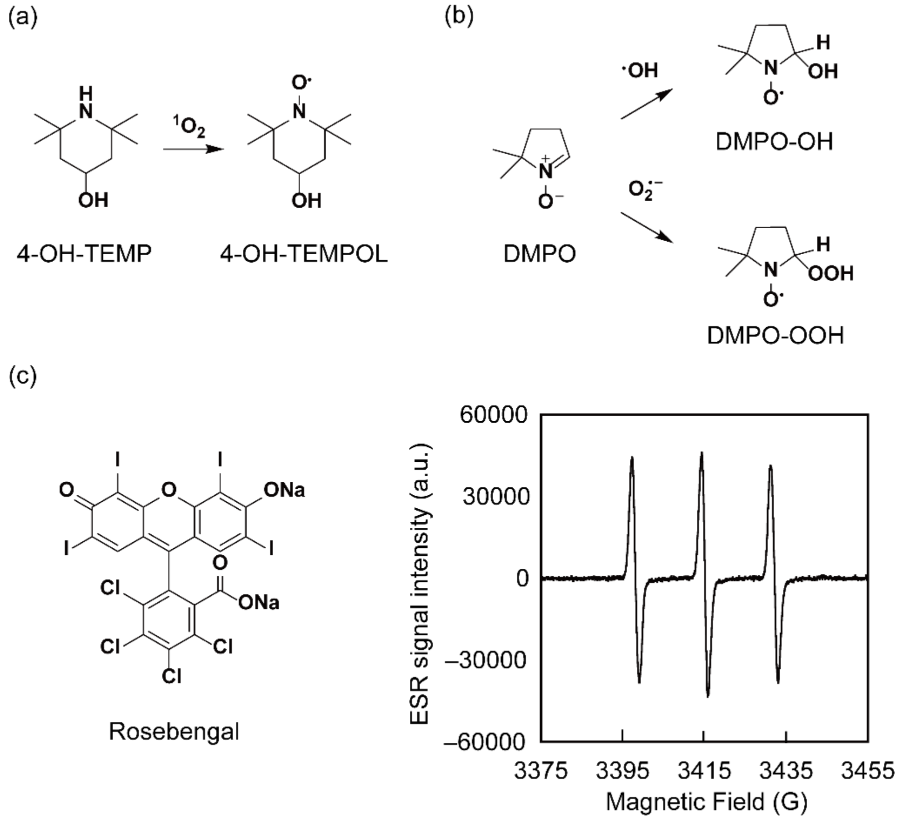

Figure S10. (a,b) Chemical structures of (a) 4-OH-TEMP and (b) DMPO, and the reactions with ROS. (c) Chemical structure of Rose Bengal and ESR spectrum of TEMPOL radical generated by the photoirradiation of Rose Bengal. Experimental conditions: [**Rose Bengal**] = 50 µM, [**4-OH-TEMP**] = 100 mM, solvent: water, rt, irradiation wavelength: 560 nm (FWHM: 10 nm, Xe lamp, 300 W, 3 min).

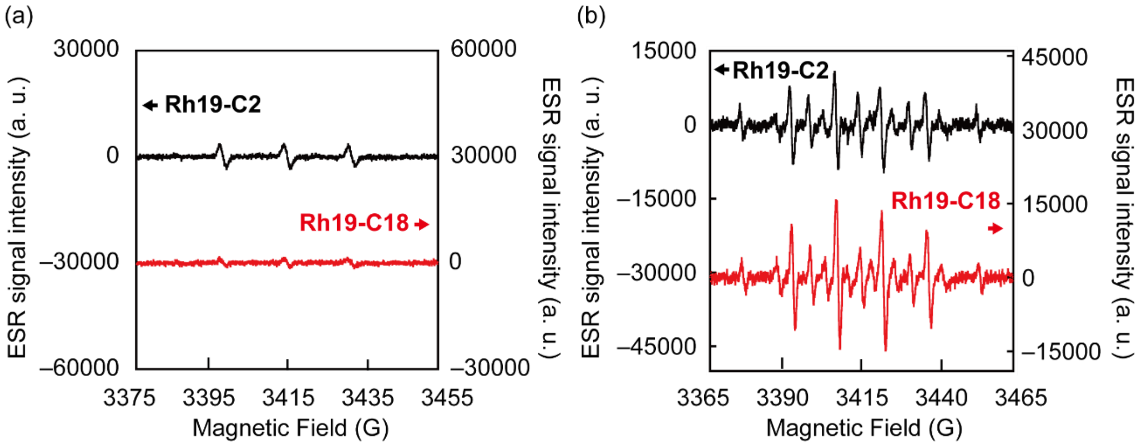

Figure S11. ESR signals of (a) TEMPOL and (b) DMPO adducts observed for the samples mixed with **Rh19-C2** and **Rh19-C18** after light irradiation. Experimental conditions: [**Rh19-C2**] = [**Rh19-C18**] = 50 μM, [4-OH-TEMP] = [DMPO] = 100 mM, solvent: water, rt, irradiation wavelength: 560 nm (FWHM: 10 nm, Xe lamp, 300 W, 3 or 10 min).

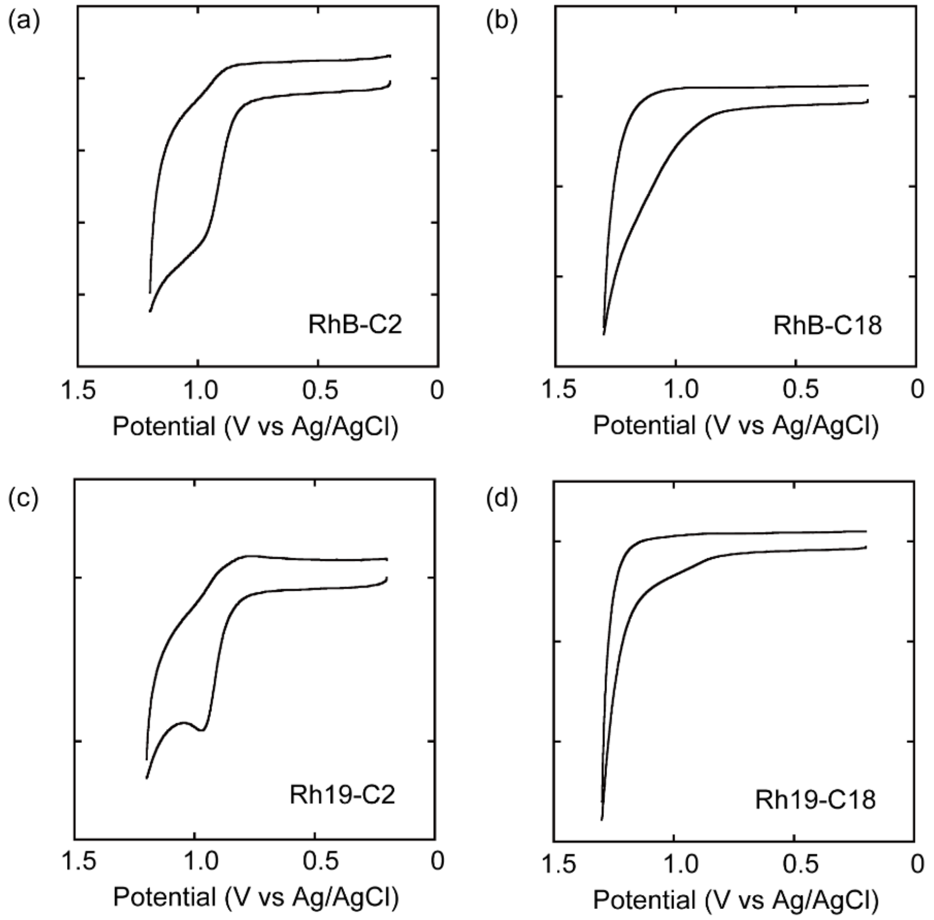

Figure S12. Cyclic voltammograms of (a) **RhB-C2**, (b) **RhB-C18**, (c) **Rh19-C2**, and (d) **Rh19-C18**.

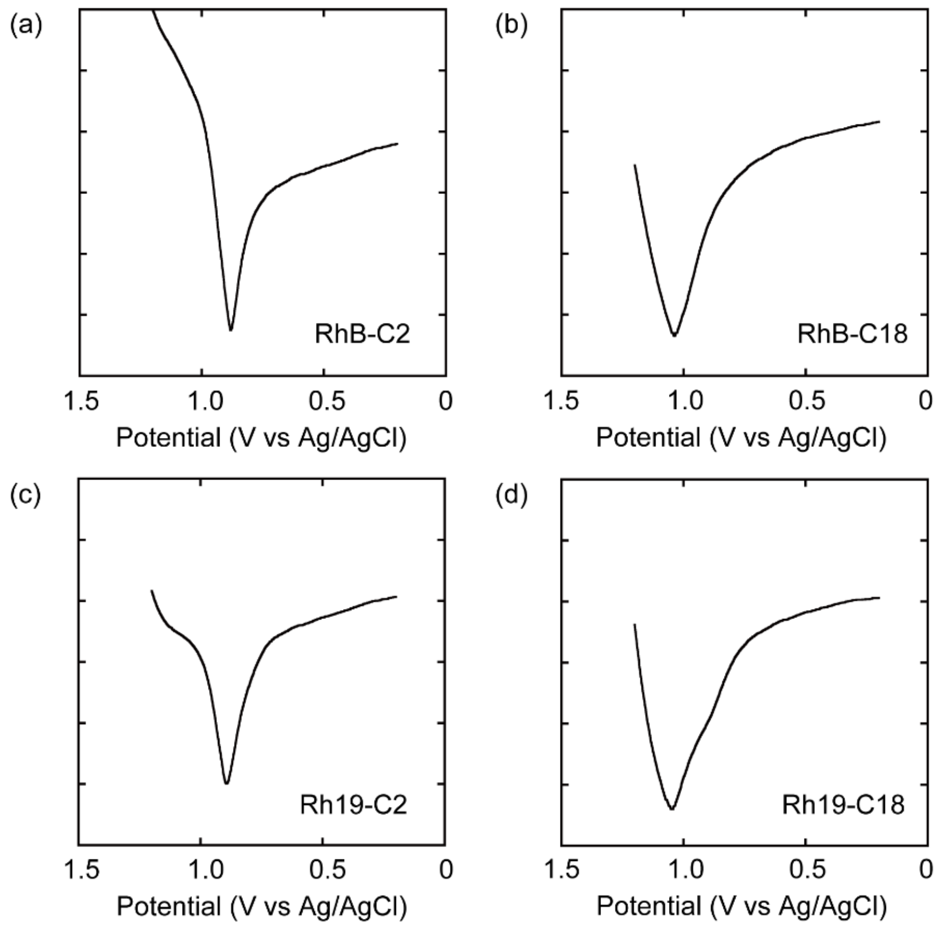

Figure S13. Squarewave voltammograms of (a) **RhB-C2**, (b) **RhB-C18**, (c) **Rh19-C2**, and (d) **Rh19-C18**.

Table S1. Optical and electrochemical properties of rhodamine derivatives.

Compound	Ox. Potential (V vs. SCE)	λ_{abs} onset (nm)	E_{0-0} (eV)	HOMO (eV)	LUMO (eV)
**RhB-C2**	0.92	585	2.12	−5.36	−3.24
**RhB-C18**	1.08	601	2.06	−5.52	−3.46
**Rh19-C2**	0.93	552	2.25	−5.37	−3.12
**Rh19-C18**	1.09	571	2.17	−5.53	−3.36

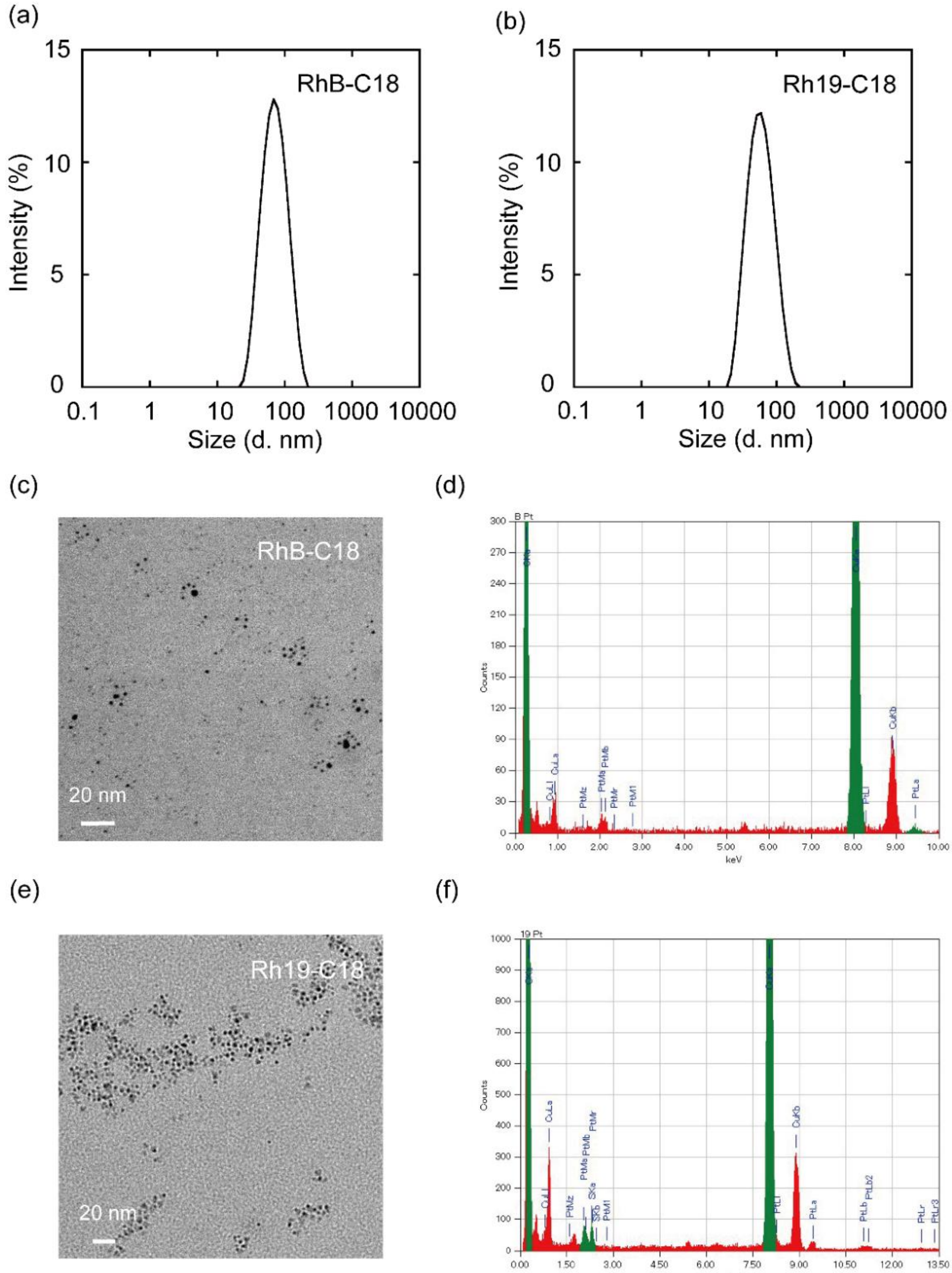

Figure S14. (a,b) Size distributions of supramolecular assemblies of (a) **RhB-C18** and (b) **Rh19-C18** determined by DLS under the presence of Asc and H_2PtCl_6 (average particle diameter: (a) 52 nm, (b) 64 nm, Polydispersity index (PDI): (a) 0.168, (b) 0.152). (c-f) TEM-EDX (energy dispersive X-ray) analysis of Pt nanoparticles of the mixed solutions. TEM images of Pt nanoparticles in the mixture of Asc, H_2PtCl_6, and (c) **RhB-C18** or (e) **Rh19-C18** after 20 min photoirradiation. (d,f) EDX spectra of selected areas ((d) **RhB-C18**, (f) **Rh19-C18**). Experimental conditions: [**RhB-C18**] = [**Rh19-C18**] = 50 μM, [Pt] = 100 μM, [Asc] = 500 mM, rt, water., Scale bar: 20 nm, irradiation light wavelength: > 360 nm, 20 min.

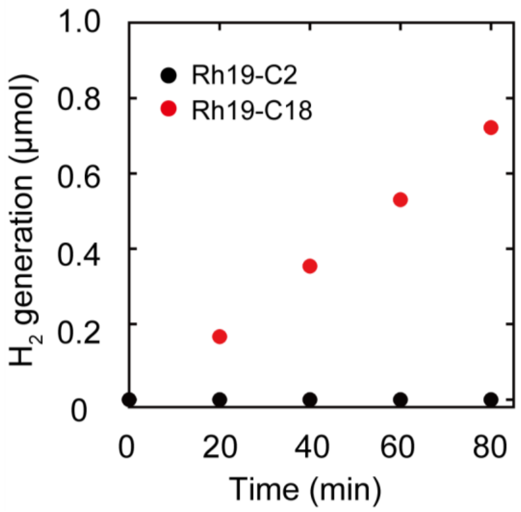

Figure S15. Time-course of hydrogen evolution by **Rh19-C2** and **Rh19-C18** under visible light. Experimental conditions: [**Rh19-C2**] = [**Rh19-C2**] = 50 μM, [Pt] = 100 μM, [Asc] = 500 mM, solvent: water, rt, irradiation light wavelength: > 360 nm.

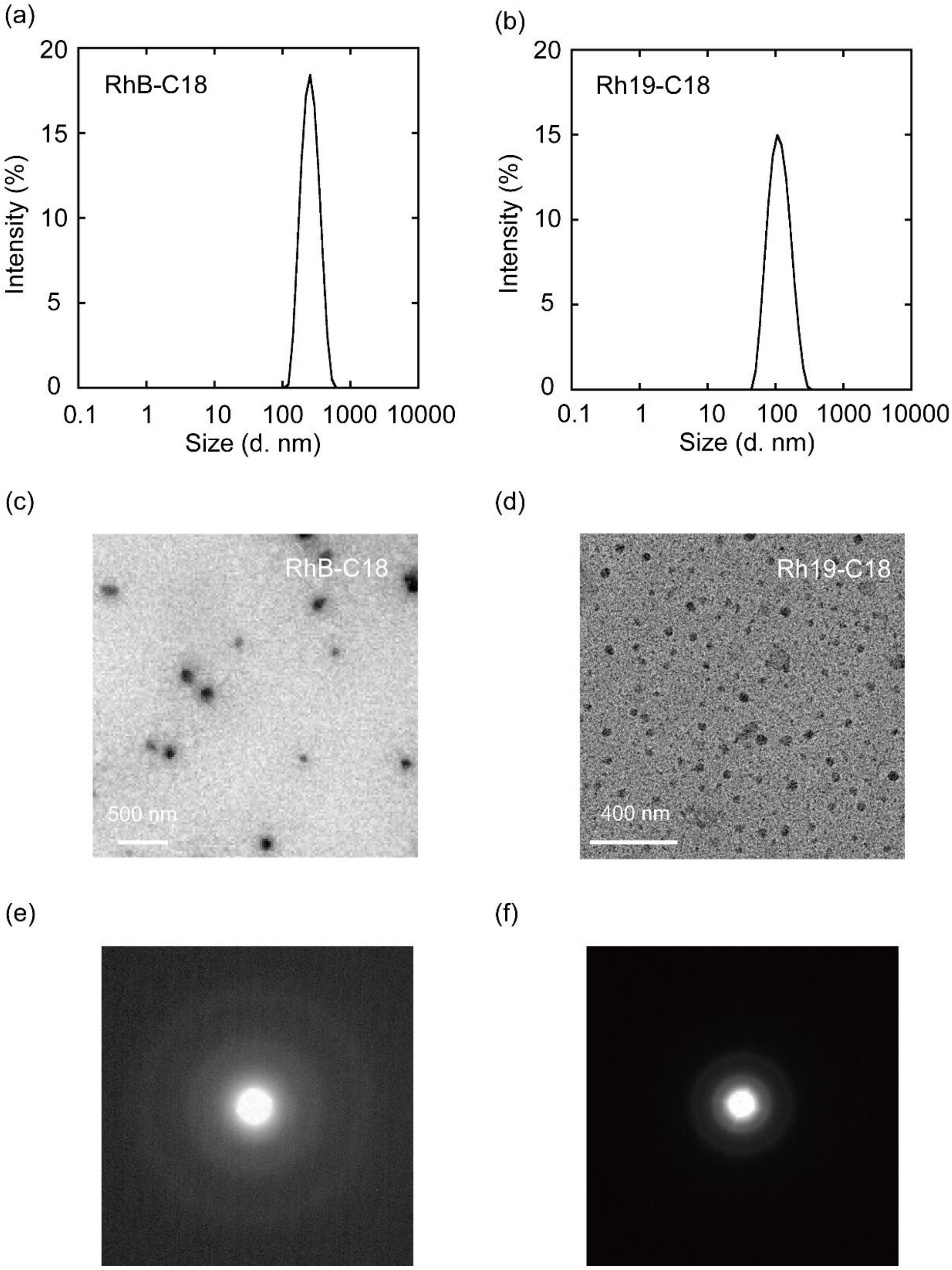

Figure S16. (a,b) Size distributions of supramolecular assemblies of (a) **RhB-C18** and (b) **Rh19-C18** determined by DLS (average particle diameter: (a) 223 nm, (b) 122 nm, Polydispersity index (PDI): (a) 0.159, (b) 0.110). (c,d) TEM images of supramolecular assemblies of (c) **RhB-C18** and (d) **Rh19-C18**. Experimental conditions: [**RhB-C18**] = [**Rh19-C18**] = 100 μM, rt, water., Scale bar: 500 nm. (e,f) SAED diffraction patterns of supramolecular assemblies of (e) **RhB-C18** and (f) **Rh19-C18**. Experimental conditions: [**RhB-C18**] = [**Rh19-C18**] = 100 μM, solvent: water.

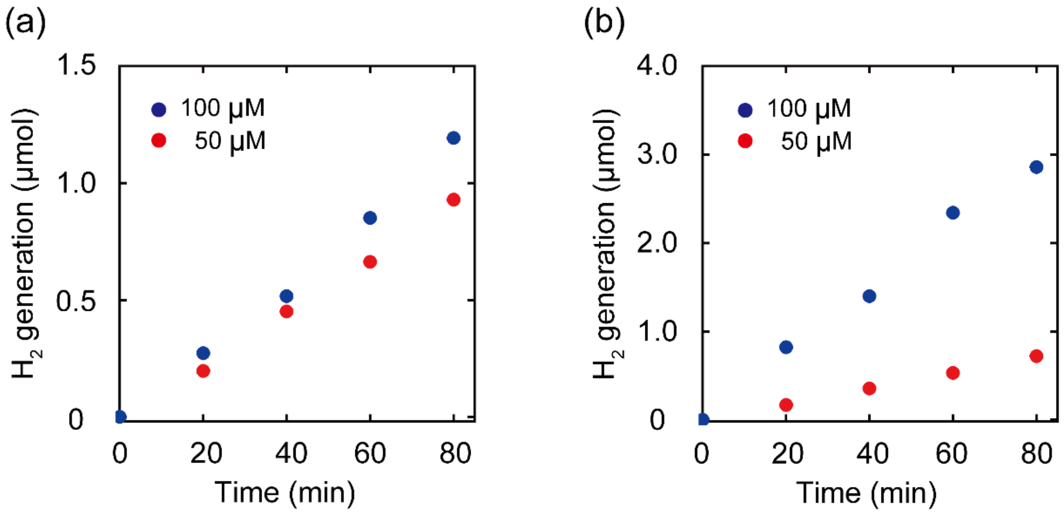

Figure S17. Time-course of hydrogen evolution by (a) **RhB-C18** and (b) **Rh19-C18** under various concentrations after irradiation of visible light. Experimental conditions: [Rhodamines] = 50 or 100 μM, [Pt] = 100 μM, [Asc] = 500 mM, solvent: water, rt, irradiation light wavelength: > 360 nm.
